# Endotypes and Phenotypes in ILD: Is It Time to Reclassify ILD by Risk Stratification?

**DOI:** 10.3390/biomedicines13071529

**Published:** 2025-06-23

**Authors:** Francesco Amati, Anna Stainer, Stefano Aliberti

**Affiliations:** 1 Department of Biomedical Sciences, Humanitas University, Pieve Emanuele, 20072 Milan, Italy; anna.stainer@hunimed.eu (A.S.); stefano.aliberti@hunimed.eu (S.A.); 2 Respiratory Unit, IRCCS Humanitas Research Hospital, Rozzano, 20089 Milan, Italy

Interstitial lung diseases (ILD) comprise a heterogeneous group of disorders that present significant challenges in both diagnosis and management due to their varied nature, which encompasses different degrees of inflammation and fibrosis affecting lung tissue [1]. The manifestation of ILD heterogeneity is multi-level and includes patients’ clinical symptoms, the trajectory of disease progression, radiological findings, histopathological characteristics, and underlying biological mechanisms [2]. This heterogeneity has been a substantial barrier to understanding disease mechanisms and developing effective and personalized treatments. Therefore, the identification and characterization of endotypes, phenotypes, and biomarkers have emerged as vital steps, not only for enhancing diagnostic accuracy, but for providing prognostic insights and facilitating tailored therapy options that can improve patient outcomes [3,4].

A prospective study showed that most of the treatable traits (TT) hypothesized in the literature based on phenotypes and endotypes could be identified in ILD cohorts [5]. This finding reinforces the critical importance of adopting a TT approach in the management of ILD. Notably, the majority of TTs observed within the ILD population were found to be related to comorbidities and extra-respiratory manifestations, thereby underscoring the necessity of a multidisciplinary team (MDT) in the comprehensive management of these patients. Establishing a systematic sequence of pharmacological and non-pharmacological interventions within the MDT framework is essential in order to adequately determine whether treatment goals should be driven by systemic disease, pulmonary disease, or both, particularly in the case of relevant comorbidities and ILD associated with connective tissue diseases (CTD) [6].

Furthermore, the dynamic nature of ILDs was emphasized through this study’s observation of changes in TTs at the one-year follow-up mark. This illustrates the importance of ongoing disease monitoring and the role of MDT discussions, not merely as diagnostic evaluations, but for ongoing management options and the identification of ILD progression. Accurately predicting disease behavior based on early-stage endotyping and phenotyping is essential, especially for ILDs in which diagnosis can be made in young adult patients and life-long treatment can be started based on the clinical picture, such as sarcoidosis or CTDs [7,8]. Identifying “high-risk patients” using biomarkers is of paramount priority, as it can help minimize patient exposure to potentially harmful treatments while promoting personalized approaches that yield better outcomes compared to currently used standard first-line therapies [9].

The papers featured in this Special Issue investigate various traits linked to biomarkers, with a particular emphasis on pulmonary fibrosis, a critical aspect of ILD that warrants focused attention.

Serum or tissue biomarker identification predicting fibrosis and its progression represents a future challenge for ILD management. One noteworthy example is HOXB7, a protein involved in regulating processes such as proliferation, motility, and angiogenesis across different neoplastic diseases [10]. Samarelli et al. showed that HOXB7 is highly expressed in the lung tissue of patients with idiopathic pulmonary fibrosis (IPF) and seems to be associated with the extension of fibrosis, suggesting its potential role as a biomarker for disease progression [11]. Additionally, HOXB7 can lead to the development and progression of IPF to lung cancer. Thus, it could be identified as a prospective biomarker for stratifying lung cancer risk among patients diagnosed with IPF.

Biomarkers are also crucial for assessing response to treatment and identifying patients exhibiting a progressive pulmonary fibrosis (PPF) phenotype. Traditionally, clinicians have relied on a combination of pulmonary function tests (PFT), radiological assessments, and clinical evaluations to monitor these factors [12]. However, PFTs measure only whole-lung physiology, representing a significant limitation in ILD, in which pathology is heterogeneously distributed. On the other hand, high-resolution computed tomography (HRCT) scans deliver excellent morphological insights into the correlates of inflammation and fibrosis while only offering limited physiological data. Quing et al. explored the role of magnetic resonance imaging using hyperpolarized 129Xe as a gaseous contrast agent (129Xe MRI) in providing regional data on lung ventilation, diffusion, and perfusion. The hyperpolarized 129Xe MRI has proven to be highly sensitive in identifying regional functional changes in ILD patients with usual interstitial pneumonia (UIP) patterns, indicating its potential as a new tool to monitor progression and response to treatment in these patients [13].

A variety of biomarkers, such as KL-6, surfactant protein D (SP-D), CXCL4, and anti-MDA5, have been linked with fibrotic progression in ILDs associated with CTD [14]. However, no singular biomarker has yet proven sufficient to predict patient PPF phenotypes. A combination of biomarkers to identify PPF patients with a “high-risk signature” could enrich clinical trial cohorts and avoid the need for antecedent progression when defining progressive fibrosing ILD for clinical trial enrolment [15].

In conclusion, the future of ILDs hinges on precision medicine approaches tailored to specific phenotypic and endotypic patient characteristics. The increasing significance of biomarkers in ILD management cannot be overstated, as they influence diagnosis, prognosis, and the personalization of treatment approaches. Recently, Alqalyoobia et al. employed high-throughput proteomics to identify and validate biomarkers associated with survival across various types of fibrotic ILD. Their findings highlighted noteworthy molecular similarities between IPF and non-IPF ILDs while also revealing unique biological signatures that could lead to more targeted therapeutic strategies [16]. The success of precision medicine in ILD will need to be verified in appropriately designed randomized controlled trials of unselected patients with ILD stratified by combinational biomarkers and/or of a patient population with defined disease endotypes [17].

## References

[1] Travis W.D., Costabel U., Hansell D.M., King T.E.J., Lynch D.A., Nicholson A.G., Ryerson C.J., Ryu J.H., Selman M., Wells A.U. (2013). An official American thoracic society/European respiratory society statement: Update of the international multidisciplinary classification of the idiopathic interstitial pneumonias. Am. J. Respir. Crit. Care Med..

[2] Kokosi M.A., Margaritopoulos G.A., Wells A.U. (2018). Personalised medicine in interstitial lung diseases. Eur. Respir. Rev..

[3] Amati F., Spagnolo P., Ryerson C.J., Oldham J.M., Gramegna A., Stainer A., Mantero M., Sverzellati N., Lacedonia D., Richeldi L. (2023). Walking the path of treatable traits in interstitial lung diseases. Respir. Res..

[4] Khor Y.H., Cottin V., Holland A.E., Inoue Y., McDonald V.M., Oldham J., Renzoni E.A., Russell A.M., Strek M.E., Ryerson C.J. (2023). Treatable traits: A comprehensive precision medicine approach in interstitial lung disease. Eur. Respir. J..

[5] Amati F., Stainer A., Maruca G., De Santis M., Mangiameli G., Torrisi C., Bossi P., Polelli V., Blasi F., Selmi C. (2024). First Report of the Prevalence at Baseline and after 1-Year Follow-Up of Treatable Traits in Interstitial Lung Diseases. Biomedicines.

[6] Ahmed S., Handa R. (2022). Management of Connective Tissue Disease-related Interstitial Lung Disease. Curr. Pulmonol. Rep..

[7] Baughman R.P., Valeyre D., Korsten P., Mathioudakis A.G., Wuyts W.A., Wells A., Rottoli P., Nunes H., Lower E.E., Judson M.A. (2021). ERS clinical practice guidelines on treatment of sarcoidosis. Eur. Respir. J..

[8] Raghu G., Montesi S.B., Silver R.M., Hossain T., Macrea M., Herman D., Barnes H., Adegunsoye A., Azuma A., Chung L. (2024). Treatment of Systemic Sclerosis-associated Interstitial Lung Disease: Evidence-based Recommendations. An Official American Thoracic Society Clinical Practice Guideline. Am. J. Respir. Crit. Care Med..

[9] Joy G.M., Arbiv O.A., Wong C.K., Lok S.D., Adderley N.A., Dobosz K.M., Johannson K.A., Ryerson C.J. (2023). Prevalence, imaging patterns and risk factors of interstitial lung disease in connective tissue disease: A systematic review and meta-analysis. Eur. Respir. Rev..

[10] Li C., Mao X., Song L., Sheng J., Yang L., Huang X., Wang L. (2024). Unveiling HOXB7 as a novel diagnostic and prognostic biomarker through pan-cancer computer screening. Comput. Biol. Med..

[11] Samarelli A.V., Tonelli R., Raineri G., Mastrolia I., Costantini M., Fabbiani L., Catani V., Petrachi T., Bruzzi G., AndrisaniDario D. (2024). Expression of HOXB7 in the Lung of Patients with Idiopathic Pulmonary Fibrosis: A Proof-of-Concept Study. Biomedicines.

[12] Raghu G., Remy-Jardin M., Richeldi L., Thomson C.C., Inoue Y., Johkoh T., Kreuter M., Lynch D.A., Maher T.M., Martinez F.J. (2022). Idiopathic Pulmonary Fibrosis (an Update) and Progressive Pulmonary Fibrosis in Adults: An Official ATS/ERS/JRS/ALAT Clinical Practice Guideline. Am. J. Respir. Crit. Care Med..

[13] Qing K., Altes T.A., Mugler J.P., Mata J.F., Tustison N.J., Ruppert K., Bueno J., Flors L., Shim Y.M., Zhao L. (2023). Hyperpolarized Xenon-129: A New Tool to Assess Pulmonary Physiology in Patients with Pulmonary Fibrosis. Biomedicines.

[14] Parker M.J.S., Jee A.S., Hansen D., Proudman S., Youssef P., Kenna T.J., Stevens W., Nikpour M., Sahhar J., Corte T.J. (2024). Multiple serum biomarkers associate with mortality and interstitial lung disease progression in systemic sclerosis. Rheumatology.

[15] Kuwana M., Avouac J., Hoffmann-Vold A.-M., Smith V., Toenges G., Alves M., Distler O. (2024). Development of a multivariable prediction model for progression of systemic sclerosis-associated interstitial lung disease. RMD Open.

[16] Alqalyoobi S., Smith J.A., Maddali M.V., Pugashetti J.V., Newton C.A., Kim J.S., Linderholm A.L., Chen C.-H., Ma S.-F., Bose S. (2025). Proteomic Biomarkers of Survival in Non-IPF Interstitial Lung Disease. Am. J. Respir. Crit. Care Med..

[17] Matson S.M., Molyneaux P.L. (2025). Blurred Boundaries: Rethinking Disease Classifications in ILD Using Molecular Signals. Am. J. Respir. Crit. Care Med..

